# Choice of Behavioral Change Techniques in Health Care Conversational Agents: Protocol for a Scoping Review

**DOI:** 10.2196/30166

**Published:** 2021-07-21

**Authors:** Laura Martinengo, Nicholas Y W Lo, Westin I W T Goh, Lorainne Tudor Car

**Affiliations:** 1 Family Medicine and Primary Care Lee Kong Chian School of Medicine Nanyang Technological University Singapore Singapore Singapore; 2 Lee Kong Chian School of Medicine Nanyang Technological University Singapore Singapore Singapore; 3 Department of Primary Care and Public Health School of Public Health Imperial College London London United Kingdom

**Keywords:** behavior change, behavioral change technique, chatbot, conversational agent, health care, protocol, scoping review, long-term outcomes, behavior

## Abstract

**Background:**

Conversational agents or chatbots are computer programs that simulate conversations with users. Conversational agents are increasingly used for delivery of behavior change interventions in health care. Behavior change is complex and comprises the use of one or several components collectively known as behavioral change techniques (BCTs).

**Objective:**

The objective of this scoping review is to identify the BCTs that are used in behavior change–focused interventions delivered via conversational agents in health care.

**Methods:**

This scoping review will be performed in line with the Joanna Briggs Institute methodology and will be reported according to the PRISMA extension for scoping reviews guidelines. We will perform a comprehensive search of electronic databases and grey literature sources, and will check the reference lists of included studies for additional relevant studies. The screening and data extraction will be performed independently and in parallel by two review authors. Discrepancies will be resolved through consensus or discussion with a third review author. We will use a data extraction form congruent with the key themes and aims of this scoping review. BCTs employed in the included studies will be coded in line with BCT Taxonomy v1. We will analyze the data qualitatively and present it in diagrammatic or tabular form, alongside a narrative summary.

**Results:**

To date, we have designed the search strategy and performed the search on April 26, 2021. The first round of screening of retrieved articles is planned to begin soon.

**Conclusions:**

Using appropriate BCTs in the design and delivery of health care interventions via conversational agents is essential to improve long-term outcomes. Our findings will serve to inform the development of future interventions in this area.

**International Registered Report Identifier (IRRID):**

PRR1-10.2196/30166

## Introduction

### Background

Conversational agents (CAs), also known as chatbots, are computer programs that simulate conversations with users [1]. CAs are multimodal and can be deployed via messaging apps such as Telegram, Facebook Messenger, and Slack [2]; as standalone apps or websites; or integrated into cars or television sets. CAs may operate using simple text interfaces as the most basic form, as voice assistants, or as embodied CAs that use virtual characters to simulate both verbal and nonverbal human behavior [3]. CAs are multifunctional and can automate a variety of tasks such as provision of information or news, web-based shopping, or as symptom checkers.

Health care is an ideal candidate for CA-delivered interventions [4]. CAs can assist patients by providing timely information [5], support mental health disorder management [6,7], and assist with triage in clinical settings [8,9] by harnessing the increasing ubiquity of smartphones [10] and other digital media [11,12].

CAs are increasingly being used for delivery of interventions focusing on disease treatment, management, and prevention [13]. An important component of such interventions is behavior change. Behavioral change interventions are defined as “coordinated sets of activities designed to change specified behavior patterns” [14]. Most behavior change interventions are complex, comprising the interaction of one or several smaller components, often referred to as behavioral change techniques (BCTs) [15]. A BCT is “an observable and replicable component designed to change behavior” [15], with descriptors as the smallest component compatible with retaining the postulated active ingredients of components designed to alter or regulate behavior, enabling easy coding and extraction of data in the intervention context [15]. Although various BCT taxonomy systems have been developed, BCT Taxonomy v1 (BCTTv1) [16] is widely used to provide a shared, standardized terminology to specify the active ingredients to support behavior change in health care interventions. BCTTv1 consists of 93 distinct BCTs divided into 19 major groups such as “goals and planning” and “feedback and monitoring” [16].

Existing reviews on CAs are largely descriptive and focus on effectiveness [17,18]. Systematic reviews on BCTs found that incorporating certain BCTs in the design of health care interventions increased their effectiveness [17-19]. Examples include action planning to enhance physical activity [20] or self-monitoring to increase physical activity and healthy eating [21]. Given the increasing use of CAs in health care and delivery of behavior change interventions, it is crucial to understand the way BCTs are incorporated in their design, including what BCTs are most commonly used and the behavior they intend to change [22]. By identifying what BCTs are employed, to what outcomes, and for what function according to experimental studies, this review seeks to advance understanding of the types of BCTs used in CAs in health care.

### Identifying the Research Question (Stage 1)

The aim of this scoping review is to identify and present BCTs that have been included in behavior change interventions delivered via CAs. Specifically, the review will try to answer the following questions: (1) Which BCTs are used in CA-delivered interventions in health care? (2) What are the target behaviors the CA in health care aims to modify? (3) What were the theories/frameworks guiding the design of behavior change interventions delivered via CAs in health care? (4) Which BCTs have been incorporated into the effective CA interventions?

## Methods

### Design

This scoping review will follow the Joanna Briggs Institute scoping review guidelines [23] and will be reported in line with the PRISMA-ScR (Preferred Reporting Items for Systematic Reviews and Meta-Analyses extension for Scoping Reviews) reporting guidelines [24].

### Identifying Relevant Studies (Stage 2)

A systematic search will be performed of both peer-reviewed and grey literature. Peer-reviewed articles will be searched on the following databases: PubMed, Embase (Ovid), and Cochrane Central Register of Controlled Trials (CENTRAL). Grey literature will be identified by searching the first 10 pages in Google and Google Scholar.

The search strategy includes an extensive list of related words and phrases that define “conversational agents,” which was updated from a search strategy used in a previous scoping review on CAs published by our research group [2] (see Multimedia Appendix 1).

All studies retrieved from the searches will be uploaded to EndNote X9 (Clarivate) and further imported to Covidence [25], an online tool to assist in the production of systematic reviews, to facilitate the screening of eligible articles. Screening will be performed in two stages, according to inclusion and exclusion criteria. In the first stage, the titles and abstracts will be screened by two independent reviewers. In the second stage, the full texts of all studies included will be acquired and reviewed by two independent investigators. Discrepancies resulting from any screening stage will be resolved by discussion between the reviewers or by engaging a third reviewer if discrepancies cannot be resolved by discussion alone. Interrater agreement will be determined using the Cohen κ coefficient in which κ≥0.6 (substantial or almost perfect agreement) will be considered to be acceptable [26]. The search and screening processes will be documented using a study selection flowchart [27].

### Study Selection (Stage 3)

This scoping review will include primary experimental studies on a CA-delivered health care intervention focusing on behavior change. The eligible study designs comprise randomized controlled trials, quasirandomized controlled trials, cluster-randomized trials, controlled before-and-after studies, uncontrolled before-and-after studies, interrupted time series, pilot, and feasibility studies. Nonexperimental study designs, including observational studies, qualitative studies, reviews, personal communications, editorials, as well as conference abstracts and studies where it was not possible to access the full text report, will be excluded. The study population will include interventions featuring any kind of CA, including a text-based, voice-based, or embodied CA. Embodied CAs are defined as conversational interfaces that include a human-like avatar mimicking human movements and facial expressions. The eligible interventions will include any health care intervention focused on behavior change. This includes interventions focusing on improving or promoting a healthy lifestyle (eg, increase physical activity, weight loss, healthy eating) or managing physical or mental health disorders (eg, depression, diabetes, asthma, hypertension). These interventions should include a clear reference to behavior change as an essential aspect of the intervention, with or without reference to an associated behavioral change theory.

### Charting the Data (Stage 4)

A data extraction form will be developed by the research team, which will include the following items: first author, year of publication, source of literature, title of article, study design and methods, geographic focus, health care sector, CA name, accessibility of CA, dialogue technique, input and output modalities, nature of the CA’s end goal, behavioral change theories or frameworks guiding the intervention, and BCTs used, mapped according to BCTTv1 [16].

Data from included studies will be extracted into a Microsoft Excel spreadsheet. Before starting the data extraction process, the table will be piloted by all members of the research team involved in this step, and amendments to the table will be made according to researchers’ feedback.

Data extraction for each paper will be performed by two researchers working independently, and the results will subsequently be compared. If disagreements arise, these will be resolved through consensus or consultation with a third reviewer acting as an arbiter.

Data will be tabulated and analyzed using descriptive statistics.

### Collating, Summarizing, and Reporting the Results (Stage 5)

The data extracted from the included papers will be presented in a diagrammatic or tabular form accompanied by a narrative summary. We will provide an overview of the choice of BCTs in the included studies, the use of BCTs across different types of CAs and health care areas, the BCTs employed in the interventions that have been shown to be effective, and the theories or frameworks used in the development of behavior change interventions. We will also discuss any gaps in the evidence and provide recommendations for future use of BCTs in CA-delivered interventions.

### Stakeholder Consultation (Stage 6)

We will also undertake a stakeholder consultation to inform the analysis and presentation of our findings. Stakeholders will include researchers in the field of CAs in health care. We will organize a seminar with the stakeholders, present our findings, and invite comments from the attendants. We will use the collated feedback to guide our analysis and reporting in our manuscript.

### Ethics and Dissemination

Ethical approval is not required for this study. We will disseminate our findings via a publication in a peer-reviewed journal and presentations at conferences.

## Results

This protocol is being submitted prior to data collection and is registered on the Open Science Framework (osf.io/487jd). To date, we have designed the search strategy (see Multimedia Appendix 1 for the PubMed search strategy), and performed the initial search in PubMed (447 articles retrieved), Embase (858 articles retrieved), CENTRAL (1003 articles retrieved), and the first 10 pages of Google and Google Scholar search results on April 26, 2021 for a total of 3303 articles. After removing duplicates, a total of 2579 articles remain for screening. The first round of screening is planned to begin shortly.

## Discussion

Behavior change is an essential yet complex aspect of many health care interventions. The aim of this scoping review is to provide insight into how behavior change interventions can be delivered via CAs. To provide a systematic and transparent analysis of this complex concept, we will identify and map the choice and use of BCTs in CA-delivered interventions. Using appropriate BCTs could potentially increase engagement and improve long-term outcomes. Our findings will serve to inform the development of future interventions in this area.

## Appendix

Multimedia Appendix 1 PubMed search strategy.

## Abbreviations

BCT: behavior change technique
BCTTv1: BCT Taxonomy v1
CA: conversational agent
CENTRAL: Cochrane Central Register of Controlled Trials
PRISMA-ScR: Preferred Reporting Items for Systematic Reviews and Meta-Analyses extension for Scoping Reviews
